# A Comprehensive Prognostic and Immunological Analysis of a New Three-Gene Signature in Hepatocellular Carcinoma

**DOI:** 10.1155/2021/5546032

**Published:** 2021-06-02

**Authors:** Jun Liu, Jianjun Lu, Wenli Li

**Affiliations:** ^1^Reproductive Medicine Center, Yue Bei People's Hospital, Shantou University Medical College, Shaoguan 512025, China; ^2^Medical Research Center, Yue Bei People's Hospital, Shantou University Medical College, Shaoguan 512025, China; ^3^The Second School of Clinical Medicine, Southern Medical University, Guangzhou 510080, China; ^4^Department of Medical Affairs, First Affiliated Hospital of Sun Yat-sen University, Guangzhou 510080, China

## Abstract

There are few reports on the role of genes associated with the mRNA expression-based stemness index (mRNAsi) in the prognosis and immune regulation of hepatocellular carcinoma (HCC). This study is aimed at analyzing the expression profile and prognostic significance of a new mRNAsi-based three-gene signature in HCC. This three-gene signature was identified by analyzing mRNAsi data from the Cancer Genome Atlas (TCGA) HCC dataset. The prognostic value of the risk score based on the three-gene signature was evaluated by Cox regression and Kaplan-Meier analysis and then verified in the International Cancer Genome Consortium (ICGC) database. Meanwhile, the correlations between the risk score and immune cell infiltration patterns, microsatellite instability (MSI), tumor mutation burden (TMB), immune checkpoint molecules, hypoxia-related genes, immunotherapy response, and compounds targeting the gene signature were explored, respectively. The results showed that compared with normal liver tissues, the mRNAsi score of HCC tissues was significantly increased. PTDSS2, MRPL9, and SOCS were the genes most related to mRNAsi in HCC tissues. Survival analysis results suggested the risk score based on the three-gene signature was an independent predictor of the prognosis for patients with HCC. The nomogram combining the risk score and pathological stage showed a good predictive ability for the overall survival of patients with HCC patients. Meanwhile, the risk score was significantly related to immune cell infiltration patterns, MSI, TMB, several immune checkpoint molecules, and hypoxia-related genes. In addition, the risk score was associated with the immunotherapy response, and fifteen potential therapeutic drugs targeting the three-gene signature were identified. Therefore, we propose to use this three-gene signature including PTDSS2, MRPL9, and SOCS as a potential prognostic biomarker for HCC.

## 1. Introduction

Hepatocellular carcinoma (HCC), which accounts for 80% -90% of all liver cancers, is the fifth most common cancer in the world and the third leading cause of all cancer-related deaths. The metastasis and recurrence of HCC make the disease difficult to cure. Although chemotherapy is a common method besides surgical resection, local ablation, and liver transplantation for patients with HCC, the high rate of drug resistance in cancer cells limits the treatment efficacy. Recently, the concept of cancer stem cells (CSC) helps to explain the metastasis, recurrence, and drug resistance of HCC. There are some CSCs in tumors, which have strong survival, growth, self-renewal, and resistance capabilities, thereby promoting tumor development and spread [1]. In the past few years, CSC was used to identify important genes in HCC [2]. It is found that CSC status can not only be used as a prognostic factor but also may be a new target for HCC treatments [3].

CSCs are generally identified by related biomarkers such as CD133, CD44s, CD90, and epithelial cell adhesion molecule (EpCAM). We have already known that CD133 (+) liver cancer cells can promote chemotherapy resistance, and increased CD133 expression is an independent prognostic factor for HCC patients. Furthermore, it is reported that the cytoplasmic expression of CD133 is significantly associated with the survival of HCC patients [4–6]. In addition, CD44s is also associated with poor prognosis in patients with HCC [7]. CD90 expression is associated with early recurrence of HCC [8]. EpCAM+ HCC cells are associated with the aggressiveness and metastasis of HCC and are closely related to overall survival [9]. Therefore, stem cell characteristics are important factors which can affect tumor recurrence and progression. The mRNAsi is an important index of CSC, and a higher mRNAsi score is associated with a greater tumor dedifferentiation. As we know, abnormal mRNA expression plays an important role in tumor biology, and some abnormal mRNAs can affect the self-renewal, proliferation, and development of CSCs. Therefore, abnormally expressed mRNAs in HCC can be used as biomarkers for evaluating the prognosis of HCC patients.

In this study, we extracted an HCC cohort from the Cancer Genome Atlas (TCGA), identified the most important prognostic genes related to mRNAsi, and established a gene signature for HCC survival prediction. The expressions of key genes were verified in the Gene Expression Omnibus (GEO) and Gene Expression Profiling Interactive Analysis (GEPIA) databases. The risk score based on the gene signature was established in the TCGA database and subsequently verified in the ICGC database. Then, the correlations between the risk score and immune cell infiltration patterns, microsatellite instability (MSI), tumor mutation burden (TMB), immune checkpoint molecules, hypoxia-related genes, and immunotherapy response were explored, respectively. In addition, we used connectivity map analysis to identify potential therapeutic compounds targeting the gene signature. Finally, we established a nomogram for clinical practice by combining the prognostic gene signature and pathological stage and then verified the prediction accuracy of the nomogram by the calibration plot, time-dependent receiver operating characteristic (ROC) curve, and decision curve analysis. In summary, in the present study, we comprehensively analyzed the prognostic and immunological significance of a new stemness-related gene signature in HCC. This gene signature can be used as a potential prognostic biomarker for HCC.

## 2. Materials and Method

### 2.1. Data Source

The training dataset with HCC-mRNA expression profiles and clinical information obtained from the TCGA database included 370 HCC tissues and adjacent non-HCC tissues (ANTTs). The validation dataset with HCC-mRNA expression profile and clinical information used to validate the multigene signature downloaded from the ICGC database included 232 HCC tissues and ANTTs. In addition, the mRNA expression profiles of ten HCC cohorts were downloaded from the GEO database to examine the mRNA expression profiles of identified key genes with prognostic significance. Meanwhile, genes were verified in the GEPIA database (http://gepia.Cancer-pku.cn) [10]. We obtained the score of immune cell infiltration from TIMER databases (https://cistrome.shinyapps.io/timer/), and tumor mutation burden of HCC was calculated based on somatic mutation. We acquired the microsatellite instability from published literature [11]. The above four databases are publicly available. Therefore, this study did not require a local ethics committee approval.

### 2.2. mRNAsi and Its Clinical Significance in HCC

CSCs are tumor cells that have a self-renew ability and play an important role in tumor survival, proliferation, metastasis, and recurrence. Stemness indices are indicators which can describe the similarity between tumor cells and stem cells. So stemness indices can be regarded as the quantification of the CSC characteristics, including mRNAsi, an index calculated based on expression data, and EREG-mRNAsi, an expression index calculated based on stem cell apparent regulation-related genes. These indices range from zero to one, and closer to one suggests a lower degree of cell differentiation and stronger characteristics of CSC. To investigate mRNAsi scores and their clinical prognostic value, we compared mRNAsi scores between HCC samples and matched normal liver tissues, observed median mRNAsi scores of different tumor grades, and compared the prognosis between patients with high mRNAsi and low mRNAsi scores.

### 2.3. Identification of Differentially Expressed Genes (DEGs) between HCC and Noncancerous Tissues

We downloaded the original sequencing data of HCC mRNA from the TCGA database and obtain DEGs (log2FC > 1 or log2FC < −1, *p* < 0.05 as a cutoff value) by the Limma R-package. The DEGs were used for subsequent analysis. The subsequent analysis methods included establishing a coexpression gene network based on RNA sequence data, identifying a gene signature with prognostic value, and verifying the independence of the gene signature as a prognostic factor followed the methods of Li et al. 2020 [12].

### 2.4. Coexpression Gene Network Based on RNA-Seq Data

We selected the DEGs identified in the previous step and performed WGCNA to construct a gene coexpression network [13]. First, we calculated the absolute value of the Pearson correlation coefficient between gene *i* and gene *j* to construct a gene expression similarity matrix:

(1) $$\begin{array}{c} S_{ij}=\left|\frac{\left(1+\text{cor}\left(x_{i}+y_{j}\right)\right)}{2}\right|, \end{array}$$

*x*_*i*_ and *y*_*j*_ represent the expression levels of gene *i* and gene *j*, respectively. Then, the gene expression similarity matrix was converted into an adjacency matrix, which increased the strong correlation and weakened the weak correlation in the exponential level. *β* is a soft threshold, which is actually the Pearson correlation coefficient of each pair of genes [14]:

(2) $$\begin{array}{c} \alpha_{\text{ij}}={\left|\frac{\left(1+\text{cor}\left({\text{x}}_{\text{i}}+{\text{y}}_{\text{j}}\right)\right)}{2}\right|}^{\beta }. \end{array}$$

Next, we converted the adjacency matrix into a topological matrix. Topological overlap measure (TOM) was used to describe the degree of association between genes:

(3) $$\begin{array}{c} \text{TOM}=\frac{\left(\sum_{\mu \neq ij}\alpha_{i\mu }\alpha_{\mu j}+\alpha_{ij}\right)}{\left(\min\;\left(\sum_{\mu }\alpha_{i\mu }+\sum_{\mu }\alpha_{j\mu }\right)+1-\alpha_{ij}\right)}. \end{array}$$

TOM indicates the difference degree between gene *i* and gene *j*. The most representative genes in each module are called feature vector genes, and ME represents the overall expression level of the genes within the module. We performed average linkage hierarchical clustering based on TOM's similarity measure, calculated their similarity, and built a module tree diagram:

(4) $$\begin{array}{c} \text{ME}=\text{princomp}\left({x_{ij}}^{q}\right). \end{array}$$

Here, *i* represents the gene in module *q*, and *j* represents the chip sample in module *q*. We used the Pearson correlation between the expression profile of genes in all samples and the ME expression profile of feature vector genes to measure the identity of the genes in the module, which was called module membership (MM):

(5) $$\begin{array}{c} {{\text{MM}}_{i}}^{q}=\text{cor}\left(x_{i},{\text{ME}}^{q}\right). \end{array}$$

Here, ME represents the expression profile of the *i* gene. We calculated the gene importance (GS) to reflect the importance of each module and used this to measure the correlation between genes and sample traits. The module importance (MS) was displayed by the average GS of the module and was used to measure the correlation between the module and sample features. In this study, we selected mRNAsi and EREG-mRNAsi as clinical phenotypes and selected modules that were significantly related to mRNAsi for subsequent analysis.

### 2.5. Definition of a Three-Gene Signature with Prognostic Values

In this study, univariate, the least absolute shrinkage and selection operator (LASSO), and multiple Cox regression analyses were used to explore the correlation between gene expression levels and the overall survival (OS). We firstly used univariate Cox regression analysis to identify OS-related genes, then applied LASSO Cox regression to further narrow the range of HCC prognostic genes, and used multiple Cox regression analysis to assess whether prognostic genes can be used as independent prognostic factors. Next, we established a prognostic gene signature by multiplying the expression coefficient of the multivariate Cox regression model (*β*) with its expression level. That is, the prognosis index (Pi) = (*β*∗PTDSS2 expression level) + (*β*∗MRPL9 expression level) − (*β*∗SOCS2 expression level). Subsequently, we divided 370 HCC patients of the TCGA into high- and low-risk groups based on the risk score derived from the prognostic model and performed Kaplan-Meier (KM) survival curves and ROC curves to assess the predictive power of the model. In addition, we validated the prognostic model in an independent HCC dataset from the ICGC database.

### 2.6. The Three-Gene Signature Is an Independent Predictor for OS in HCC

We used univariate and multivariate Cox regression analysis to assess whether the three-gene signature could be used as a risk factor independent of other clinical pathological variables such as age, sex, tumor grade, and pathological stage for HCC patients. We took clinical characteristics as independent variables, OS as the dependent variable, and calculated the hazard ratio (HR) (95% confidence interval, two-sided *p* value).

### 2.7. Enrichment Analysis of Genes in the High-Risk Score Group

HCC samples from the TCGA were divided into high- and low-risk groups based on the expression level of the three-gene signature. Then, set enrichment analysis (GSEA) was performed using GSEA software (https://www.broadinstitute.org/gsea/) to find pathways in genes of the high-risk group enriched. Relevant settings were alignment = gene set, metric = _class difference, #arrange = 2500.

### 2.8. Validation of the Three-Gene Signature by an Independent HCC Dataset

We verified the prognostic value of the three-gene signature in an independent HCC dataset from the ICGC database. Taking the median risk score of all HCC patients in the validation dataset as a cutoff value, we divided HCC patients with follow-up information into high- and low-risk groups and compared the OS between the two groups (two-sided *p* value, and *p* < 0.05 represents a significant statistical difference).

### 2.9. Correlation Analysis between Risk Score and Tumor Immune Microenvironment (TIME)

TIME is an immune-related complex environment for tumor cells to survive and develop. In order to evaluate the interaction between risk score and TIME, we analyzed the correlations between the risk score and immune cell infiltration patterns, MSI, TMB, immune checkpoint, and hypoxia-related genes, respectively. Subsequently, we analyzed the survival rate of HCC patients from the above aspects (two-factor analysis). The immune infiltrating cells included in this study were CD8 T cells, B cells, dendritic cells, CD4 T cells, neutrophils, and macrophages. The immune checkpoint molecules included in the analysis were PDCD1, CTLA4, CD80, CD86, CD274, PDCD1LG2, CD276, and VTCN1. The hypoxia-related genes included in the analysis were obtained from previous literature, including SLC2A1, LDHA, ALDOA, ENO1, VEGFA, ACOT7, TPI1, CDKN3, MRPS17, MIF, NDRG1, TUBB6, ADM, PGAM1, and PGAM1 [15, 16].

### 2.10. Quantification of the Risk Score as an Immune Response Predictor Using Immunophenoscore

Immunophenoscore (IPS), which is based on major histocompatibility complex-related molecules, checkpoints/immunomodulators, effector cells, and suppressor cells, can be used to quantify the determinants of tumor immunogenicity and characterize intratumor immune landscapes and cancer antigenomes. The sum of the weighted average *Z*-scores calculated using the sample *Z*-scores of each category is called IPS [17]. In this study, IPS was used to predict the response of anti-CTLA-4 and anti-PD-1 regimens.

### 2.11. Prediction of Potential Therapeutic Compounds Using Connectivity Map Analysis

Using connectivity map analysis, we identified compounds targeting the three-gene signature that may lead to novel treatments that trigger differentiation and exhaust the stemness potential of neoplasms [18].

### 2.12. Establishment and Evaluation of the Nomogram for Survival Prediction of HCC

The nomogram is a simplified OS assessment diagram that converts statistical prediction models into diagrams suitable for clinical use. In this study, we combined the risk score based on the three-gene signature and pathological stage to construct a nomogram that can assess the 1-, 3-, and 5-year survival probabilities of HCC patients and compared the predicted probability of the nomogram with the observed actual survival probability by a calibration curve to verify the accuracy of the nomogram. The overlap of the lines indicates that the model is accurate. Besides, the ROC curve was used to evaluate the prediction accuracy of the nomogram.

## 3. Results

### 3.1. Entire Study Process and Summary of Patients' Information

**Figure 1:**
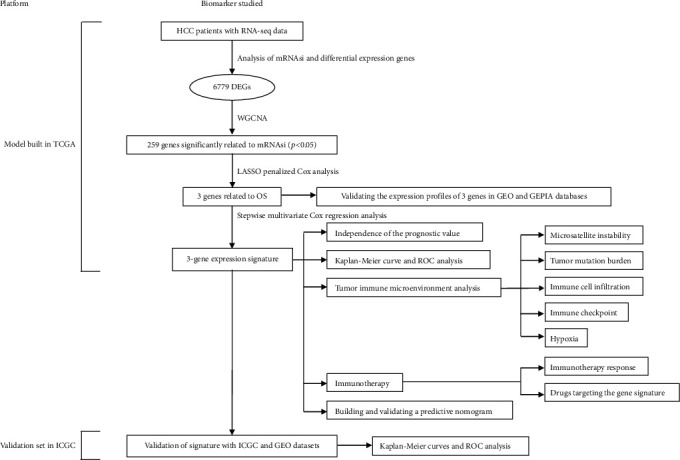
Overall flowchart of this study.

Figure 1 is a flowchart of the entire research work. This figure shows the detailed construction process of an integrated model for predicting the overall survival of HCC patients based on a multigene signature through a network analysis of the transcriptome data stemness indices. The patients' information in the TCGA and ICGC cohorts is shown in Table 1.

### 3.2. Network Analysis of mRNAsi and DEGs in HCC

**Figure 2:**
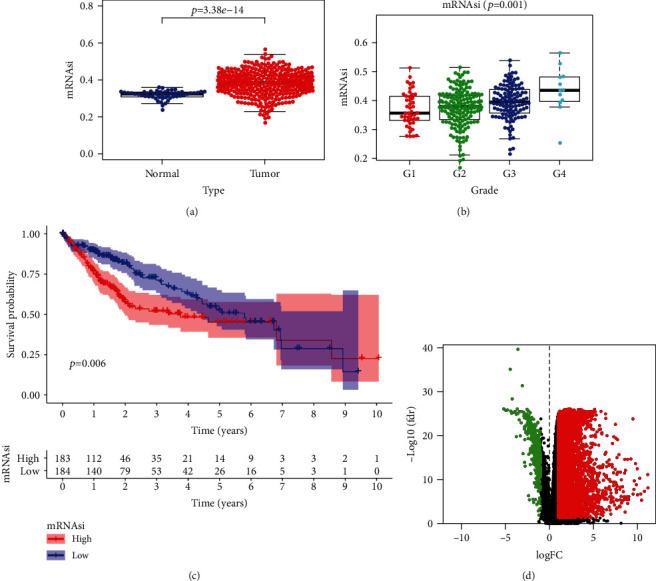
Stemness-characteristic index and its prognostic significance in HCC. (a) Differences in mRNAsi between HCC and matched normal liver tissues in the TCGA dataset. (b) Comparison of mRNAsi among HCC subgroups divided by pathological grades. (c) Kaplan-Meier curves of patients assigned to high- and low-risk groups based on the mRNAsi value in the TCGA dataset. The prognosis of the high-mRNAsi group was poorer than that of the low-mRNAsi group. (d) Volcano plot showing DEGs in HCC samples. Green indicates downregulated of genes, and red indicates upregulated of genes.

Cancer cell stemness is of clinical importance, and mRNAsi is a quantitative expression of CSC. In this study, the HCC dataset from the TCGA database was downloaded to analyze mRNAsi scores in HCC tissues. As shown in Figure 2. there was a significant difference in mRNAsi scores between the HCC tissues and adjacent nontumor tissue (ANTTs) (Figure 2(a)), and mRNAsi scores increased with increasing HCC grade (Figure 2(b)). In addition, patients with higher mRNAsi scores had a poorer prognosis than patients with lower mRNAsi scores (Figure 2(c)). In order to compare the mRNA expression profiles of tumor tissues and normal tissues, the HCC expression matrix from the TCGA database was downloaded to screen DEGs, and 6,779 DEGs were screened out (log2FC > 1 or log FC < −1, *p* < 0.05), of which 6356 were upregulated and 423 were downregulated (Figure 2(d)).

### 3.3. Identification of the Most Significant Modules Associated with mRNAsi

In this study, important genetic modules related to the stemness of cancer cells were identified by WGCNA. We selected 6779 differential genes for WGCNA processing, constructed gene coexpression modules, and assigned these genes to different modules through the cluster tree diagram (Figure S1A). The gene numbers of each module in WGCNA were shown in Table 2. It was found that the modules with a higher coexpression correlation coefficient with mRNAsi were the black, blue, and yellow-green modules (correlation coefficients were -0.59, 0.50, and 0.47, respectively). The correlation coefficient between each coexpressed gene module and HCC stemness (mRNAsi and EREG-mRNAsi) was shown in Figure S1B. The results of modules correlation analysis suggested that there were high correlations among the black, blue, and green-yellow gene modules (Figure S1C). And the associations between these gene modules and phenotypes were the most significant (Figure S1D). Therefore, the black, blue, and green-yellow modules were considered to be the most important modules associated with mRNAsi.

### 3.4. Constructing a Three-Gene Signature for Survival Prediction

In order to establish a clinical survival prediction model for HCC based on CSC, we used a HCC cohort from the TCGA database as the training dataset and applied LASSO Cox regression analysis to identify stable markers from 259 survival-related candidates. We reduced some coefficients to zero by forcing the sum of the absolute values of the regression coefficients to be less than a fixed value. Next, we used the relative regression coefficients to determine the most stable prognostic indicators and performed cross-validation to avoid overfitting the LASSO Cox model (Figure S2A). In the established Cox model, two filter markers including PTDSS2 and MRPL9 were associated with high risk (HR > 1), and one filter marker SOCS2 was associated with low risk (HR < 1) (Figure S2B).

Then, we applied the above 3 genes to construct a multigene signature based on the minimum criteria for the survival prediction of HCC. We used the coefficients obtained from the Cox regression analysis to calculate the risk score for each HCC patient in the training set. In order to test the relationship between the three-gene signature and the prognosis of HCC patients, we established a prognosis model based on the three-gene signature: Prognostic index = 0.236923∗PTDSS2 + 0.534246∗MRPL9 − 0.50027∗SOCS2. Then, taking the median risk score of all HCC patients in the training dataset as a cutoff value, we divided 370 HCC patients with follow-up information into high-risk (*n* = 185) and low-risk (*n* = 185) groups and compared the gene expression profiles and survival status of the two groups. The results suggested that compared with the low-risk group, the high-risk group had a worse prognosis. In addition, the expression levels of MRPL9 and PTDSS2 were higher, while the expression level of SOCS2 was lower in the high-risk group, compared with those in the low-risk group (Figure S2C).

Next, we verified the survival prediction ability of the three-gene signature in an independent HCC cohort from the ICGC database. We extracted the mRNA expression profile data and follow-up information of 232 HCC patients from this validation set and then calculated the risk score of each HCC patient using the same formula as the training set. Similarly, taking the median of the risk score as a cutoff value, we divided 232 HCC patients into high-risk group (*n* = 116) and low-risk group (*n* = 116) and compared the gene expression profiles and survival status of the two groups. The results showed that compared with the low-risk group, the high-risk group had a worse prognosis. In addition, the expression levels of MRPL9 and PTDSS2 in the high-risk group were higher, while the expression level of SOCS2 was lower, compared with those in the low-risk group. These results were consistent with that in the training set, thus, further verifying the expression profile and prognostic value of the three-gene signature (Figure S2D).

### 3.5. Kaplan-Meier and ROC Analyses of the Three-Gene Signature

**Figure 3:**
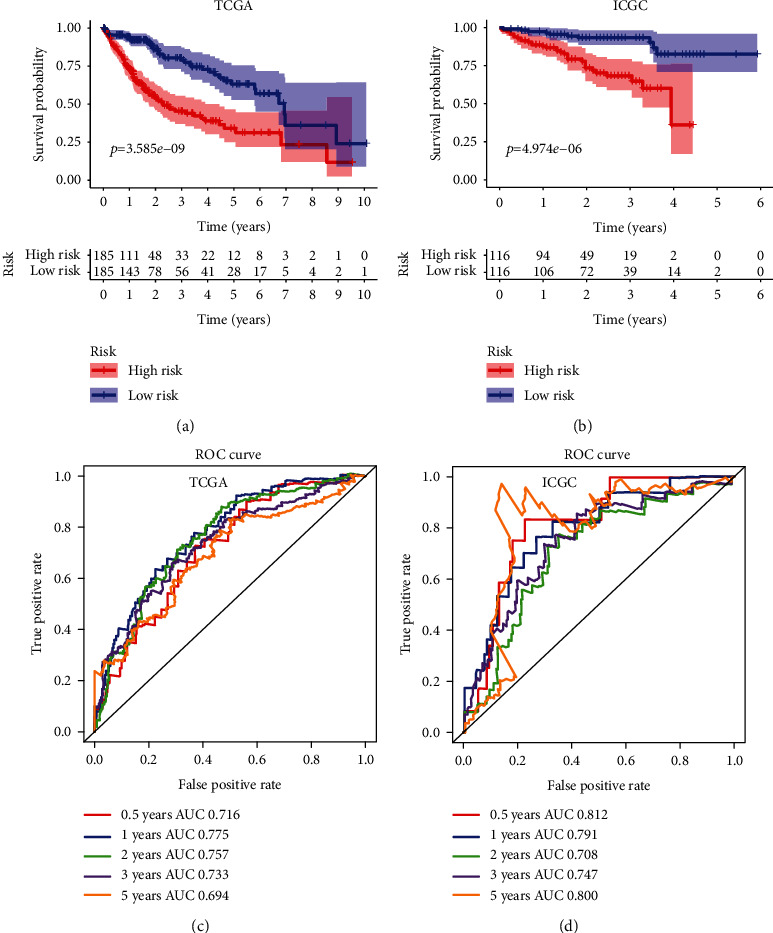
Survival analysis with the three-gene signature in the training and validation datasets. (a and b) Kaplan-Meier overall survival analysis of HCC patients in the TCGA (a) and ICGC (b) datasets assigned to high-and low-risk groups based on the risk score. Patients with a higher risk score exhibited poorer overall survival in the training and validation cohorts. (c and d) ROC curves showed the predictive efficiency of the risk signature for HCC patients in the TCGA (c) and ICGC (d) datasets on the survival rate.

Subsequently, we used the median risk score as a cutoff value to divide HCC patients into high- and low-risk groups and compared the overall survival of the two groups using the Kaplan-Meier survival curves. In addition, we used a time-dependent ROC curve to evaluate the predictive capacity of the three-gene signature. A higher AUC in the ROC curve means better prediction model performance. As shown in Figure 3(a), there was a significant difference in OS between the high- and low-risk groups in the TCGA cohort (*p* < 0.0001). The AUCs of the risk scores corresponding to 0.5, 1, 2, 3, and 5-year survival were 0.716, 0.775, 0.757, 0.733, and 0.694, respectively, indicating that the prediction model had high sensitivity and specificity (Figure 3(c)). As shown in another Kaplan-Meier curve (Figure 3(b)), the OS in the low-risk group was significantly better compared to the high-risk group in an independent validation HCC cohort from the ICGC (*p* < 0.001). This result was consistent with our previous findings in the training cohort from the TCGA dataset. As shown in Figure 3(d), the AUCs of the risk scores corresponding to 0.5, 1, 2, 3, and 5-year survival were 0.812, 0.791, 0.708, 0.747, and 0.800, respectively, which further confirms that the three-gene signature was with high sensitivity and specificity and can be used as a reliable predictor of OS in HCC.

### 3.6. Risk Score Was an Independent Prognostic Factor from the Other Clinicopathological Features

**Figure 4:**
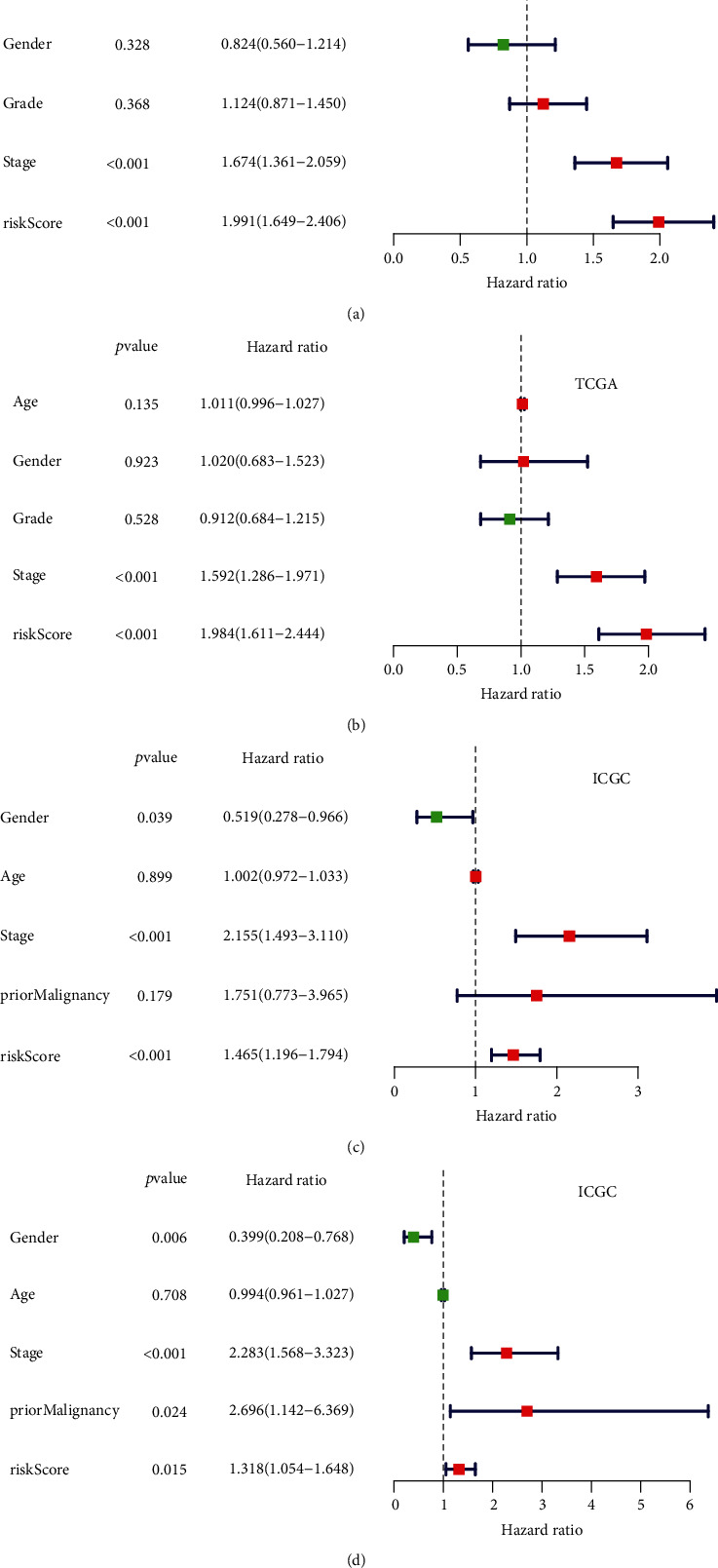
Cox regression analysis of the association between clinicopathological features and the overall survival of HCC patients. Univariate/multivariate Cox regression analyses of the association between clinicopathological factors (including the risk score) and overall survival of patients in the TCGA (a and b) and ICGC (c and d) datasets.

Univariate and multivariate Cox regression analyses were used to assess whether the risk score based on the three-gene signature could be used as an independent prognostic indicator for OS prediction in HCC patients. In the TCGA dataset, the results of both univariate and multiple Cox regression analysis suggested that the risk score and pathological stages were significantly related to OS, while age, gender, and histological grade were not related to OS (Figures 4(a) and 4(b)). In the ICGC dataset, the results of univariate Cox analysis suggested that risk score, gender, and pathological stage were significantly related to OS (Figure 4(c)). And the results of multivariate Cox regression analysis showed that risk score, gender, previous malignant tumors, and pathological stages were significantly related to OS (Figure 4(d)). These results confirmed that the risk score based on the three-gene signature can be used as an independent predictor of prognosis in patients with HCC.

### 3.7. Subgroup Analysis Based on Various Grouping Methods about Clinical Characteristics

As shown in Figure S3, in order to further verify the accuracy of risk scoring model based on the three-gene signature, we classified the HCC patients based on factors such as age, pathological stage, and histological grade. Then, we conducted subgroup survival analysis in the TCGA and ICGC datasets, respectively. The results proved that the risk score based on the three-gene signature was a biomarker for predicting OS in different subgroups, including TNM stage I-II (*p* < 0.001), stage III-IV (*p* < 0.001), G1 & G2 (*p* < 0.001), G3 & 4 (*p* < 0.001), age < 60 (*p* < 0.001), and age > 60 (*p* < 0.001) in the TCGA dataset, and TNM stage I-II (*p* = 0.019), stage III-IV (*p* = 0.025), age < 60 (*p* = 0.001), and age > 60 (*p* < 0.001) in the ICGC dataset.

### 3.8. Validating the Prognostic Value of Three-Gene Signature in an Independent HCC Cohort

We further validated the prognostic value of the risk scores based on the three-gene signature in an independent HCC cohort from the GEO database. We extracted the mRNA expression profile data and follow-up information of 209 HCC patients from this validation cohort and then calculated the risk score of each HCC patient using the same formula as the training set. Likewise, the median risk was used as a cutoff value. HCC patients were divided into the high-risk group (*n* = 104) and low-risk group (*n* = 105), and the gene expression profiles and the overall survival rates of the two groups were compared. The results suggested that the prognosis of the high-risk group was poorer (*p* < 0.01), with higher expression of MRPL93 and PTDSS2, and lower expression of SOCS2 (Figure S4A-B). These results further validated our analysis results in the training set. Subsequently, we used the ROC curve to evaluate the predictive ability of the risk score. As shown in Figure S4C, the AUCs of the risk scores corresponding to 0.5, 1, 2, 3, and 5-year survival were 0.723, 0.639, 0.673, 0.640, and 0.654, respectively, indicating that the prediction model had high sensitivity and specificity.

### 3.9. Biomarker Performance of the Three-Gene Signature in HCC

In order to evaluate the performance of PTDSS2, MRPL9, and SOCS2 as prognostic biomarkers, first, we verified the differential expression profiles of the three identified genes in HCC tissues and normal liver tissues in multiple HCC series of the GEO database. The results showed that there were significant differences in the expression levels of PTDSS2, MRPL9, and SOCS2 between HCC tissues and matched normal liver tissues (Table S1, Table S2, and Table S3). Meanwhile, we examined the expression trends of these three genes in different pathological stages of HCC in the GEPIA dataset. The expression levels of PTDSS2 and MRPL9 were higher in HCC tissues of more advanced TNM stage (*p* < 0.05; Figure S5A-B), while the expression level of SOCS2 was lower in more advanced TNM stage (*p* < 0.05; Figure S5C). Besides, we used the Kaplan-Meier curves to examine the association between the expressions of three key genes and OS, respectively. We divided HCC patients into high- and low-expression groups according to the median expression value of each identified gene. The results suggested that the high expressions of PTDSS2 (HR = 2.1, *p* < 0.0001; Figure S5D) and MRPL9 (HR = 1.8, *p* = 0.0016; Figure S5E) were associated with poorer prognosis, while lower expression of SOCS2 was associated with poorer prognosis (HR = 0.41, *p* < 0.0001; Figure S5F).

In addition, we divided the HCC samples from the TCGA database into high- and low-risk groups by the risk scores calculated based on the expression profile of the three-gene signature and used the GSEA to explore the pathways in which genes of the high-risk group enriched. The results indicated that the upregulated genes were mainly involved in the pathways of spliceosome, cell cycle, bladder cancer, DNA replication, RNA degradation, and proteasome (Figure S6). The detailed parameters related to the upregulation of signaling response genes in HCC were showed in Table S4.

### 3.10. Correlations between the Risk Score and Immune Cell Infiltration Patterns, TMB, and MSI with Corresponding Two-Factor Analysis on Survival Prediction

**Figure 5:**
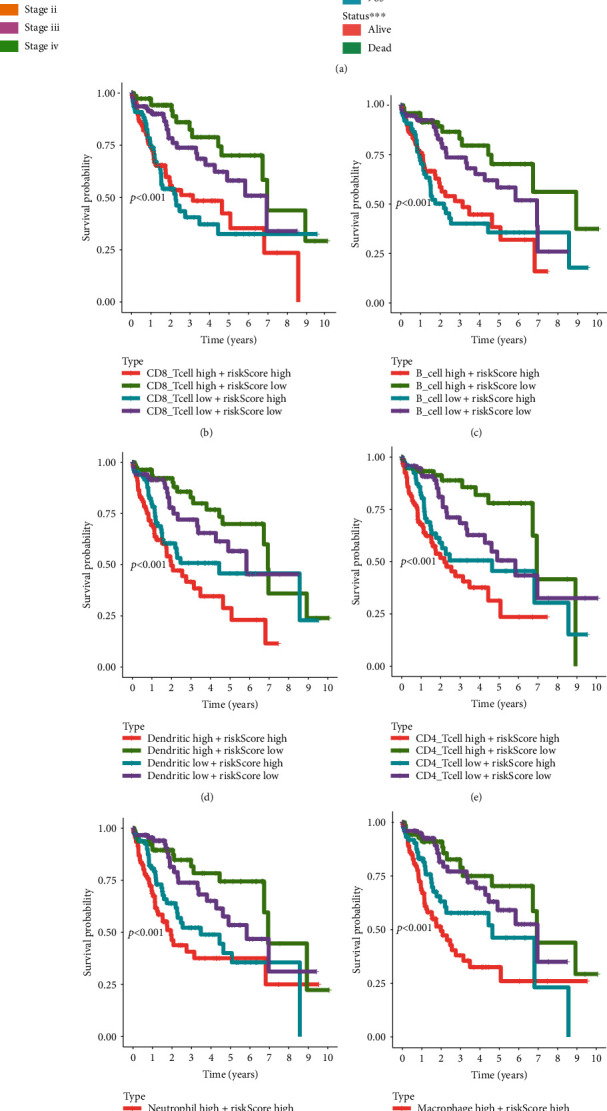
Survival analysis of HCC patients based on the risk score, immune cell infiltration patterns, MSI, and TMB. (a) Correlation analysis between risk score and immune cell, TMB, and MSI. (b–i) Two-factor survival analyses of risk score and immune cell infiltration indicators including CD8 T cell (b), B cell (c), dendritic (d), CD4 T cell (e), neutrophil (f), macrophage (g), MSI (h), and TMB (i). MSI: microsatellite instability; TMB: tumor mutation burden.

Heat maps and Kaplan-Meier analysis were performed to detect the correlation between the risk score and immune cell infiltration with clinical manifestations. The results showed that the risk score was related to immune cell infiltration, MSI, and TMB (Figure 5(a)). Combined risk score and immune cells score analyses showed that HCC patients with low-risk scores and high CD8 T cell, B cell, dendritic, CD4 T cell, neutrophil, or macrophage cell scores showed the best OS (Figures 5(b)–5(g)). The group with high-risk scores and low CD8+ T cells or low B cells scores showed a worse prognosis (Figures 5(b) and 5(c)). It is known that tumor-associated macrophage cells are negatively correlated with clinical outcomes. Unsurprisingly, the group combining high-risk scores and high macrophage cell scores showed the worst OS (Figure 5(g)). TMB and MSI are related to genome instability. And the results showed that combined low-risk scores and low TMB or MSI scores showed a better prognosis, while combined high-risk scores and high TMB or MSI scores showed a poorer prognosis.

### 3.11. Correlations between the Risk Score and Immune Checkpoints with Corresponding Two-Factor Analysis on Survival Prediction

**Figure 6:**
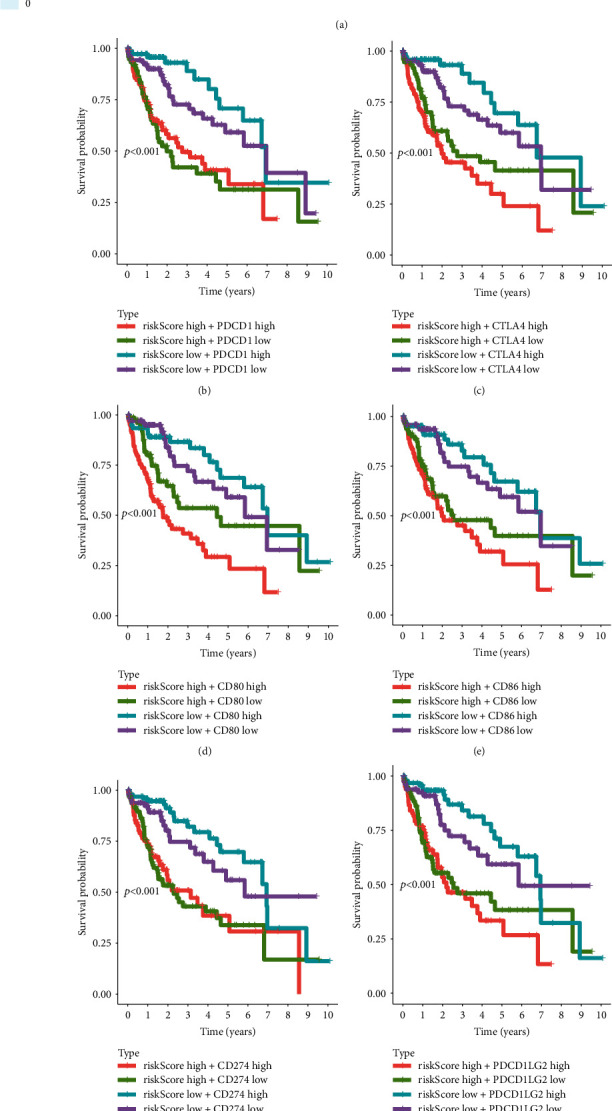
Survival analysis of HCC patients based on the risk score and several immune checkpoint molecules. (a) Correlation analysis between risk score and several key immune-related molecules. (b–i) Two-factor survival analyses of risk score and immune checkpoint molecules including PDCD1 (b), CTLA4 (c), CD80 (d), CD86 (e), CD274 (f), PDCD1LG2 (g), CD276 (h), and VTCN1 (i).

Immune checkpoints refer to a series of molecules expressed on immune cells that can regulate the degree of immune activation and play an important role in preventing the occurrence of autoimmunity. Therefore, heat maps and Kaplan-Meier analysis were performed to detect the correlation between the risk score and immune checkpoint molecules (including PDCD1, CTLA4, CD80, CD86, CD274, PDCD1LG2, CD276, and VTCN1) with clinical manifestations. The results showed that the risk score was associated with the expression of these immune checkpoint molecules (Figure 6(a)). Combined risk score and immune checkpoint molecules expressions analysis showed that regardless of the expression of immune checkpoint molecules, HCC patients with high-risk scores showed a worse prognosis. Besides, the group with low-risk score and high expressions of PDCD1, CTLA4, CD80, CD86, CD274, PDCD1LG2, CD276, or VTCN1 showed the best prognosis (Figures 6(b)–6(i)).

### 3.12. Correlations Analysis between the Risk Score and Hypoxia-Related Genes with Corresponding Two-Factor Analysis on Survival Prediction

**Figure 7:**
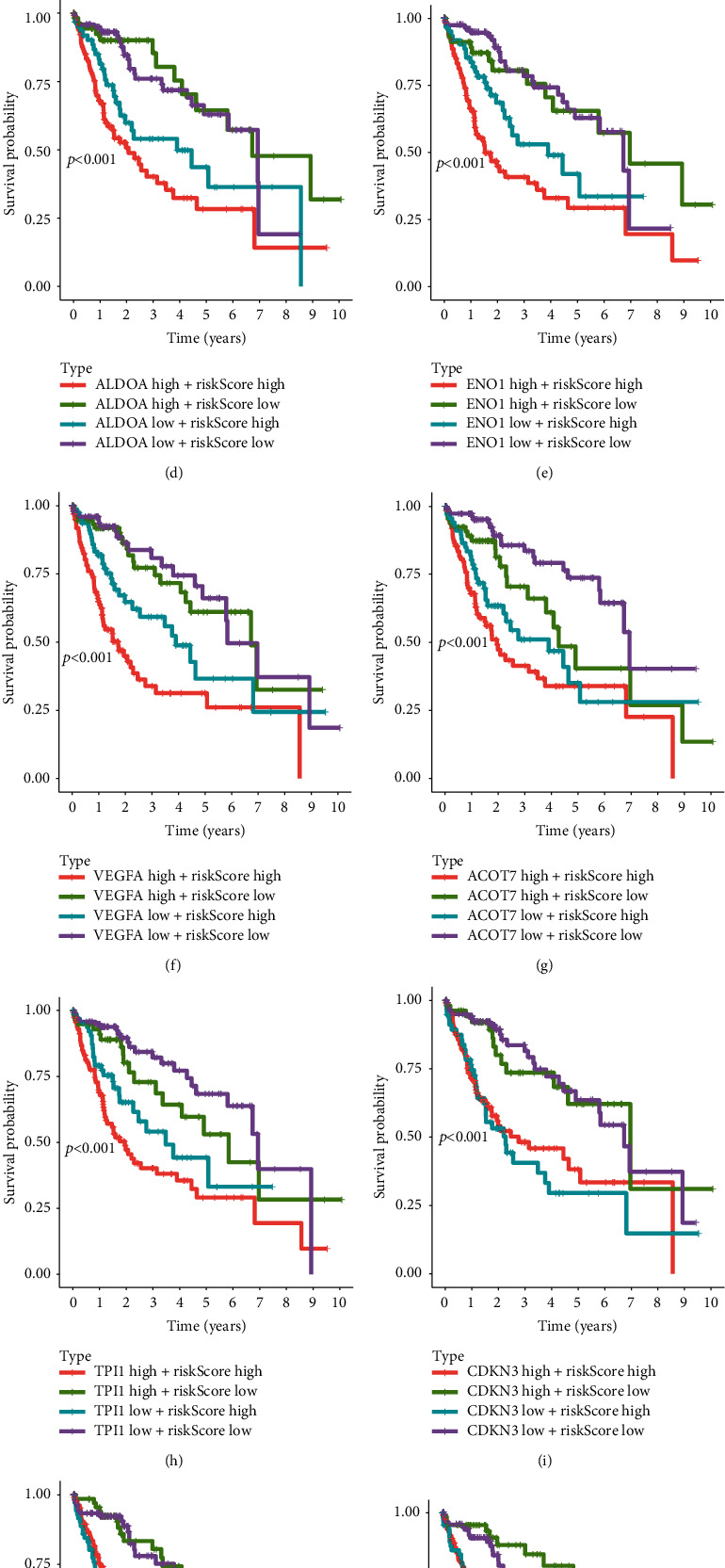
Survival analysis of HCC patients based on the risk score and several hypoxia-related genes. (a) Expression profiles of several hypoxia-related genes in high-risk and low-risk groups. (b–l) Two-factor survival analyses of risk score and immune checkpoint molecules including SLC2A1 (b), LDHA (c), ALDOA (d), ENO1 (e), VEGFA (f), ACOT7 (g), TPI1 (h), CDKN3 (i), MRPS17 (j), MIF (k), and NDRG1 (l).

Hypoxia is an important factor in the formation of TIME. We obtained 15 genes related to hypoxia in TIME from previous literature and analyzed the expression of these 15 genes in the high- and low-risk groups. The results suggested that the expressions of SLC2A1, LDHA, ALDOA, ENO1, VEGFA, ACOT7, TPI1, CDKN3, MRPS17, MIF, and NDRG1 in the high-risk group were significantly higher than that in the low-risk group, while the expressions of TUBB6, ADM, PGAM1, and PGAM1 were not significantly different from that in the low-risk group (Figure 7(a)). Combined risk score and hypoxia-related genes expressions analysis showed that regardless of the expressions of hypoxia genes, the group with high risk-score and high expressions of SLC2A1, LDHA, ALDOA, ENO1, VEGFA, ACOT7, or TPI1 presented the worst prognosis (Figures 7(b)–7(h)), while the expressions of CDKN3, MRPS17, MIF, and NDRG1 had no significant effect on the prognosis (Figures 7(i)–7(l)).

### 3.13. The Role of Risk Score in Predicting the Benefit of Immunotherapy

The treatment of ICI represented by CTLA-4/PD-1 inhibitors has made important progress in antitumor therapy. Predictors such as TML, PD-L1, and IPS are widely used to assess immune response [19]. Our analysis shows that in the CTLA-4(+) & PD-1(+), CTLA-4(+) & PD-1(-), and CTLA-4(-) & PD-1(+) immunotherapy cohorts, IPS in the low-risk group was significantly increased (*p* < 0.001, Figure S7A-C). These findings indirectly proved that the three-gene signature played an important role in mediating the immune response. Therefore, associating the risk score with the immunotherapy response can further predict the patient's prognosis.

### 3.14. Connection Diagram Analysis Identifies Potential Compounds Capable of Targeting the Three-Gene Signature

Connection map (CMap) is a data-driven method for discovering the correlation among genes, chemicals, and biological conditions. Therefore, we used the CMap to search for candidate compounds that may target the identified stemness-related gene signature. The results suggested that fifteen compounds related to stemness were significantly enriched (Table S5). These compounds may have the effect of inhibiting tumors related to stemness and were potential drugs for targeting tumors.

### 3.15. Building a Nomogram to Predict OS in HCC Patients

**Figure 8:**
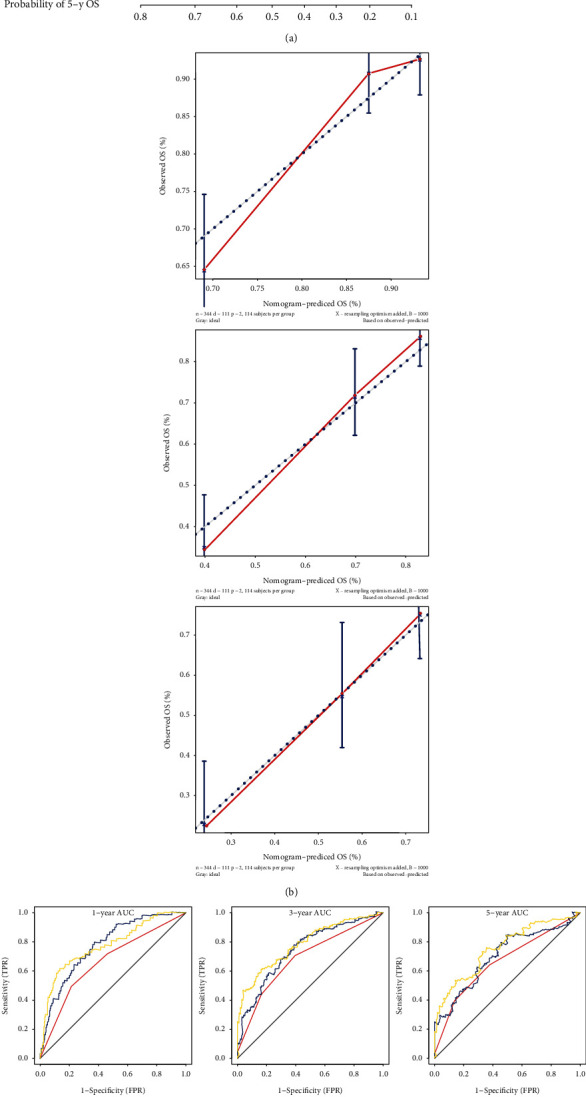
Constructing a nomogram based on the three-gene signature for survival prediction. (a) The nomogram combining the three-gene signature with clinicopathological features. (b) Calibration plot showing that nomogram-predicted survival probabilities corresponded closely to the actual observed proportions. (c) ROC curves showed the predictive efficiency of the stage model, risk score model, and combined model on 1-year, 3-year, and 5-year survival prediction in the validation HCC cohort. (d) Decision curve analysis showed the relations between net benefit and threshold probability for 1-year, 3-year, and 5-year survival prediction.

In order to establish a clinically applicable method for predicting the survival rate of patients with HCC, we built a nomogram combining the risk score and pathological stage to predict the 1-, 3-, and 5-year survival probability of patients with HCC (Figure 8(a)). Then, we analyzed the model accuracy by the calibration curves. The results showed that the 1-, 3-, and 5-year survival probabilities predicted by the nomogram were closely related to the observed survival probability, which confirmed the reliability of the nomogram (Figure 8(b)).

Then, time-based ROC curve was used to evaluate the prediction accuracy of the nomogram. The solid yellow line represents this combined model. As shown in Figure 8(c), the AUC of the nomogram that combines pathological stage and risk score was the largest, and all the AUCs of nomogram predicts for 1-, 3-, and 5-year survival prediction were above 0.75, which indicated that the nomogram constructed by integrating multiple prognostic factors was a better model to predict the survival rate of HCC patients compared to the model constructed by a single prognostic factor. In addition, as shown in Figure 8(d), we plotted the calculated net benefit against the threshold probability of patients with 1-, 3-, and 5-year survival rates. These results showed that the net benefit of the nomogram was better than other models.

## 4. Discussion

CSCs play an important role in the metastasis and recurrence of HCC [20]. Therefore, a comprehensive prognostic model based on mRNAsi, a quantitative indicator of CSCs, can more accurately predict the overall survival of HCC patients.

In this study, we focused on the construction of a prognostic model of HCC based on the three-gene signature related to the cancer cell stemness. First, the results of survival analysis suggested that HCC patients with higher mRNAsi scores had a worse prognosis than HCC patients with lower mRNAsi scores. Then, three modules significantly related to mRNAsi were identified by WGCNA. The blue and yellow-green modules were positively correlated with mRNAsi, while the black module was negatively correlated with mRNAsi. Next, all genes in the above three modules were analyzed by univariate, LASSO, and multiple regression analysis to further screen out key genes with prognostic value. PTDSS2, MRPL9, and SOCS2 were subsequently identified. It is worth mentioning that the results of interactive verification using GEO and GEPIA databases suggested that PTDSS2 and MRPL9 were overexpressed in HCC tissues, while that of SOCS2 was decreased in HCC tissues. And higher expression levels of PTDSS2 and MRPL9, as well as lower expression levels of SOCS2, were associated with worse prognosis of HCC patients. Subsequently, the results of survival analysis using the TCGA and ICGC databases confirmed that the risk score based on the three-gene signature was an independent predictor of the prognosis in HCC. Meanwhile, the risk score was significantly associated with immune cell infiltration patterns, MSI, TMB, several immune checkpoint molecules, and hypoxia-related genes. Besides, the risk score was related to immunotherapy response and fifteen compounds targeting the three-gene signature were identified. Finally, we combined the risk score and pathological stage to construct a nomogram for clinical practice. The calibration plot, ROC curve, and decision curve analysis showed that the nomogram had a good ability to predict the OS of HCC patients.

Pathological staging is a commonly used method to assess the prognosis of HCC patients, but its accuracy is easily affected by the clinical heterogeneity of HCC. The predictive ability of HCC survival prediction models based on prognosis-related biomarkers is superior to the pathological stage. Identification of prognostic-related biomarkers is the basis for establishing multigenic biomarkers. Due to the important role of cancer stem cells in tumor progression, genetic biomarkers based on cancer stem characteristics may have better predictive capacity. In fact, several stemness-related biomarkers have been reported until now. These biomarkers include mRNAs and noncoding RNAs, for example, the hypoxic response factor Artemin, which plays an important role in hypoxia-induced CSC amplification [21]. Sox2 is significant for the self-renewal of CSCs and is a predictor of poor prognosis for HCC patients after liver resection [22]. Sox9 is necessary for maintaining stem characteristics of CSCs [23]. Overexpressions of SALL4 and Cripto-1 are associated with a poorer prognosis [24, 25]. In terms of noncoding RNAs, both microRNAs and lncRNAs are verified in playing a role in the acquisition and maintenance of stem characteristics in HCC. For example, miR-137 is a prognostic biomarker for HCC patients with HCC [26]. The downexpression of miR-25 can promote the apoptosis of liver cancer stem cells by the PTEN/PI3K/Akt/Bad signaling pathway [27]. MiR-106b-5p can promote the stemness maintenance in HCC [28]. Overexpression of miR-150 can lead to cycle arrest and cell apoptosis of CD133 (+) HCC cells and negatively regulate CD133 (+) HCC stem cells [29]. Besides, long non-coding RNAs such as lnc-DILC and lncRNA-DANCR are potential stemness-related prognostic biomarkers in HCC [30, 31]. In addition, there are potential correlations between circular RNAs and CSCs [32].

Although many single genes can be considered as prognostic biomarkers of HCC, many experts believe that clinicians should be careful to use individual biomarkers as a single prognostic parameter [33]. It is reported that although CD90 and OCT4 are independent and reliable biomarkers for predicting the prognosis of HCC patients after liver resection surgery, the combination of the two biomarkers can better predict the prognosis of HCC than using any one biomarker alone [34]. In addition, PTEN combined with the expression of CD133 or EpCAM can better monitor the recurrence and predict prognosis in HCC [35].

The results of our study suggested that the three-gene signature including PTDSS2, MRPL9, and SOCS was of good prognostic value. PTDSS2 is a protein-coding gene involved in the biosynthesis and metabolic pathways of glycerophospholipids. MRPL9 is a protein-coding gene involved in mitochondrial translation and viral mRNA translation pathways. And SOCS2 is a protein-coding gene involved in the IL10 signal transduction pathway. Previous studies suggest that silencing SOCS2 can promote the progression of HCC, while the roles of PTDSS2 and MRPL9 in HCC have not been fully understood yet [36]. At the same time, the enrichment analysis results in this study suggested that the enrichment pathways of the high-risk score group based on three-gene signature included spliceosomes, cell cycle, bladder cancer, DNA replication, RNA degradation, and proteasome. These results suggest that the mechanisms of PTDSS2, MRPL9, and SOCS2 involved in tumor progression deserve further study.

In recent years, tumor immunotherapy has become a hot spot in the field of tumor therapy, and TIME plays an important role in suppressing or enhancing immune response. Therefore, gene signatures related to TIME and immunotherapy response are not only important biomarkers to exploring the mechanism of tumor occurrence and development but also are expected to provide new methods for improving the therapeutic effects of current immunotherapy. As far as we know, this new three-gene signature associated with cancer cell stemness, TIME, and immunotherapy response could be used as a new biomarker which enriches the assessment methods of survival prediction for HCC patients. Nevertheless, further studies are required to clarify the molecular mechanism involved in this three-gene signature.

## 5. Conclusion

In summary, we identified a new three-gene signature including PTDSS2, MRPL9, and SOCS which can be used as a potential prognostic biomarker for HCC. Besides, the nomogram based on the three-gene signature is a reliable tool which could help clinicians develop more personalized treatment for HCC patients.

## Figures and Tables

**Table 1:** Patients' information in the TCGA and ICGC cohorts.

Clinical characteristics		Total	%
TCGA		370	
Survival status	Survival	244	65.95
	Death	126	34.05
Age	≤65years	232	62.70
	>65 years	138	37.30
Gender	Male	249	67.30
	Female	121	32.70
Histological grade	G1	55	14.86
	G2	177	47.84
	G3	121	32.70
	G4	12	3.24
Stage	I	171	46.22
	II	85	22.97
	III	85	22.97
	IV	5	1.35
T classification	T1	181	48.92
	T2	93	25.14
	T3	80	21.62
	T4	13	3.51
	TX	1	0.27
M classification	M0	266	71.89
	M1	4	1.08
	MX	100	27.03
N classification	N0	252	68.11
	N1	4	1.08
	NX	113	30.54
ICGC		232	
Survival status	Survival	189	81.47
	Death	43	18.53
Age	≤65 years	90	38.79
	>65 years	142	61.21
Gender	Male	171	73.71
	Female	61	26.29
Stage	I	36	15.52
	II	106	45.69
	III	71	30.60
	IV	19	8.19
Prior malignancy	No	202	87.07
	Yes	30	12.93

**Table 2:** Gene numbers of each module in WGCNA.

Module	Gene number
Black	94
Blue	421
Brown	250
Green	119
Kelly	60
Grey	3062
Magenta	74
Pink	89
Purple	71
Red	108
Tan	55
Turquoise	2206
Yellow	170

## Data Availability

The datasets analyzed in the current study are available in the TCGA repository (http://cancergenome.nih.gov/), the ICGC (https://icgc.org/), and the GEO (https://www.ncbinlm. nih. gov/geo/).

## Supplementary Materials

Supplementary Materials See Figures S1 in the Supplementary Materials for the weighted gene coexpression network with DEGs between HCC and matched normal tissues. See Figures S2 in the Supplementary Materials for the risk score based on the three-gene signature is a prognostic biomarker. See Figures S3 in the Supplementary Materials for the survival analysis of different subgroups divided by clinicopathological factors. See Figures S4 in the Supplementary materials for an independent HCC cohort from the GEO database was used to verify the prognostic value of the risk score based on the three-gene signature. See Figures S5 in the Supplementary Materials for the Kaplan-Meier survival analysis with the mRNA expression of PTDSS2, MRPL9, and SOCS2. See Figures S6 in the Supplementary materials for enriched pathways of upregulated genes identified by GSEA in HCC. See Figures S7 in the Supplementary materials for the benefit of immunotherapy related to the risk score. See Table S1-S3 in the Supplementary Materials for the comparisons of PTDSS2, MRPL9, and SOCS2 expression levels between HCC and matched adjacent nontumor tissues in the GEO database, respectively. See Table S4 in the Supplementary Materials for the enriched pathways of upregulated genes using GSEA in HCC. See Table S5 in the Supplementary Materials for the potential therapeutic compounds from the CMap for the high-risk group.

## References

[1] Ma S. (2013). Biology and clinical implications of CD133 (+) liver cancer stem cells. *Experimental Cell Research*.

[2] Tong C. M., Ma S., Guan X. Y. (2011). Biology of hepatic cancer stem cells. *Journal of Gastroenterology and Hepatology*.

[3] Fan S. T., Yang Z. F., Ho D. W. Y., Ng M. N. P., Yu W. C., Wong J. (2011). Prediction of posthepatectomy recurrence of hepatocellular carcinoma by circulating cancer stem cells a prospective study. *Annals of Surgery*.

[4] Song W., Li H., Tao K. (2008). Expression and clinical significance of the stem cell marker CD133 in hepatocellular carcinoma. *International Journal of Clinical Practice*.

[5] Ma S., Lee T. K., Zheng B. J., Chan K., Guan X. Y. (2008). CD133^+^ HCC cancer stem cells confer chemoresistance by preferential expression of the Akt/PKB survival pathway. *Oncogene*.

[6] Sasaki A., Kamiyama T., Yokoo H. (2010). Cytoplasmic expression of CD133 is an important risk factor for overall survival in hepatocellular carcinoma. *Oncology Reports*.

[7] Mima K., Okabe H., Ishimoto T. (2012). CD44s regulates the TGF-*β*–mediated mesenchymal phenotype and is associated with poor prognosis in patients with hepatocellular carcinoma. *Cancer Research*.

[8] Guo Z., Li L. Q., Jiang J. H., Ou C., Zeng L. X., Xiang B. D. (2014). Cancer stem cell markers correlate with early recurrence and survival in hepatocellular carcinoma. *World Journal of Gastroenterology*.

[9] Sun Y. F., Xu Y., Yang X. R. (2013). Circulating stem cell-like epithelial cell adhesion molecule-positive tumor cells indicate poor prognosis of hepatocellular carcinoma after curative resection. *Hepatology*.

[10] Yang L., Xu Y., Yan Y. (2019). Common nevus and skin cutaneous melanoma: prognostic genes identified by gene co-expression network analysis. *Genes (Basel)*.

[11] Cortes-Ciriano I., Lee S., Park W. Y., Kim T. M., Park P. J. (2017). A molecular portrait of microsatellite instability across multiple cancers. *Nature Communications*.

[12] Li W., Lu J., Ma Z., Zhao J., Liu J. (2019). An integrated model based on a six-gene signature predicts overall survival in patients with hepatocellular carcinoma. *Frontiers in Genetics*.

[13] Langfelder P., Horvath S. (2008). WGCNA: an R package for weighted correlation network analysis. *BMC Bioinformatics*.

[14] Horvath S., Zhang B., Carlson M. (2006). Analysis of oncogenic signaling networks in glioblastoma identifies ASPM as a molecular target. *Proceedings of the National Academy of Sciences of the United States of America*.

[15] Buffa F. M., Harris A. L., West C. M., Miller C. J. (2010). Large meta-analysis of multiple cancers reveals a common, compact and highly prognostic hypoxia metagene. *British Journal of Cancer*.

[16] Zhang Q., Huang R., Hu H. (2020). Integrative analysis of hypoxia-associated signature in pan-cancer. *iScience*.

[17] Charoentong P., Finotello F., Angelova M. (2017). Pan-cancer immunogenomic analyses reveal genotype-immunophenotype relationships and predictors of response to checkpoint blockade. *Cell Reports*.

[18] Malta T. M., Sokolov A., Gentles A. J. (2018). Machine learning identifies stemness features associated with oncogenic dedifferentiation. *Cell*.

[19] Chen H., Yang M., Wang Q., Song F., Li X., Chen K. (2019). The new identified biomarkers determine sensitivity to immune check-point blockade therapies in melanoma. *Onco Immunology*.

[20] Liu C. G., Liu L. M., Chen X. J. (2018). LSD1 stimulates cancer-associated fibroblasts to drive Notch3-dependent self-renewal of liver cancer stem-like cells. *Cancer Research*.

[21] Zhang M., Zhang W., Wu Z. (2016). Artemin is hypoxia responsive and promotes oncogenicity and increased tumor initiating capacity in hepatocellular carcinoma. *Oncotarget*.

[22] Huang P. Z., Qiu J. L., Li B. K. (2011). Role of Sox2 and Oct4 in predicting survival of hepatocellular carcinoma patients after hepatectomy. *Clinical Biochemistry*.

[23] Liu C. G., Liu L. M., Chen X. J. (2016). Sox9 regulates self-renewal and tumorigenicity by promoting symmetrical cell division of cancer stem cells in hepatocellular carcinoma. *Hepatology*.

[24] Oikawa T., Kamiya A., Zeniya M. (2013). Sal-like protein 4 (SALL4), a stem cell biomarker in liver cancers. *Hepatology*.

[25] Lo R. C. L., Leung C. O. N., Chan K. K. S. (2018). Cripto-1 contributes to stemness in hepatocellular carcinoma by stabilizing dishevelled-3 and activating Wnt/*β*-catenin pathway. *Cell Death and Differentiation*.

[26] Sakabe T., Azumi J., Umekita Y. (2017). Prognostic relevance of miR-137 in patients with hepatocellular carcinoma. *Liver International*.

[27] Feng X. N., Jiang J. J., Shi S. H., Xie H. Y., Zhou L., Zheng S. S. (2016). Knockdown of miR-25 increases the sensitivity of liver cancer stem cells to TRAIL-induced apoptosis via PTEN/PI3K/Akt/Bad signaling pathway. *International Journal of Oncology*.

[28] Shi D. M., Bian X. Y., Qin C. D. (2018). miR-106b-5p promotes stem cell-like properties of hepatocellular carcinoma cells by targeting PTEN via PI3K/Akt pathway. *Oncotargets and Therapy*.

[29] Zhang J., Luo N., Luo Y., Peng Z. P., Zhang T. (2012). microRNA-150 inhibits human CD133-positive liver cancer stem cells through negative regulation of the transcription factor c-Myb. *International Journal of Oncology*.

[30] Wang X., Sun W., Shen W. F. (2016). Long non-coding RNA DILC regulates liver cancer stem cells via IL-6/STAT3 axis. *Journal of Hepatology*.

[31] Yuan S. X., Wang J., Yang F. (2016). Long noncoding RNA DANCR increases stemness features of hepatocellular carcinoma by derepression of CTNNB1. *Hepatology*.

[32] Zhu Y. J., Zheng B., Luo G. J. (2019). Circular RNAs negatively regulate cancer stem cells by physically binding FMRP against CCAR1 complex in hepatocellular carcinoma. *Theranostics*.

[33] Salnikov A. V., Kusumawidjaja G., Rausch V. (2009). Cancer stem cell marker expression in hepatocellular carcinoma and liver metastases is not sufficient as single prognostic parameter. *Cancer Letters*.

[34] Zhao R. C., Zhou J., Chen K. E. (2016). The prognostic value of combination of CD90 and OCT4 for hepatocellular carcinoma after curative resection. *Neoplasma*.

[35] Su R. J., Nan H. C., Guo H. (2016). Associations of components of PTEN/AKT/mTOR pathway with cancer stem cell markers and prognostic value of these biomarkers in hepatocellular carcinoma. *Hepatology Research*.

[36] Chen M., Wei L., Law C. T. (2018). RNA N6-methyladenosine methyltransferase-like 3 promotes liver cancer progression through YTHDF2-dependent posttranscriptional silencing of SOCS2. *Hepatology*.

