# Supplementary material for: A Comprehensive Prognostic and Immunological Analysis of a New Three-Gene Signature in Hepatocellular Carcinoma
Source: Stem Cells Int. 2021 Jun 2;2021:5546032. doi: 10.1155/2021/5546032 (PMC8192212; doi:10.1155/2021/5546032)
Supplement: Supplementary Materials — See Figures S1 in the Supplementary Materials for the weighted gene coexpression network with DEGs between HCC and matched normal tissues. See Figures S2 in the Supplementary Materials for the risk score based on the three-gene signature is a prognostic biomarker. See Figures S3 in the Supplementary Materials for the survival analysis of different subgroups divided by clinicopathological factors. See Figures S4 in the Supplementary materials for an independent HCC cohort from the GEO database was used to verify the prognostic value of the risk score based on the three-gene signature. See Figures S5 in the Supplementary Materials for the Kaplan-Meier survival analysis with the mRNA expression of PTDSS2, MRPL9, and SOCS2. See Figures S6 in the Supplementary materials for enriched pathways of upregulated genes identified by GSEA in HCC. See Figures S7 in the Supplementary materials for the benefit of immunotherapy related to the risk score. See Table S1-S3 in the Supplementary Materials for the comparisons of PTDSS2, MRPL9, and SOCS2 expression levels between HCC and matched adjacent nontumor tissues in the GEO database, respectively. See Table S4 in the Supplementary Materials for the enriched pathways of upregulated genes using GSEA in HCC. See Table S5 in the Supplementary Materials for the potential therapeutic compounds from the CMap for the high-risk group. [file 5546032.f1.docx]

Supplementary Figures


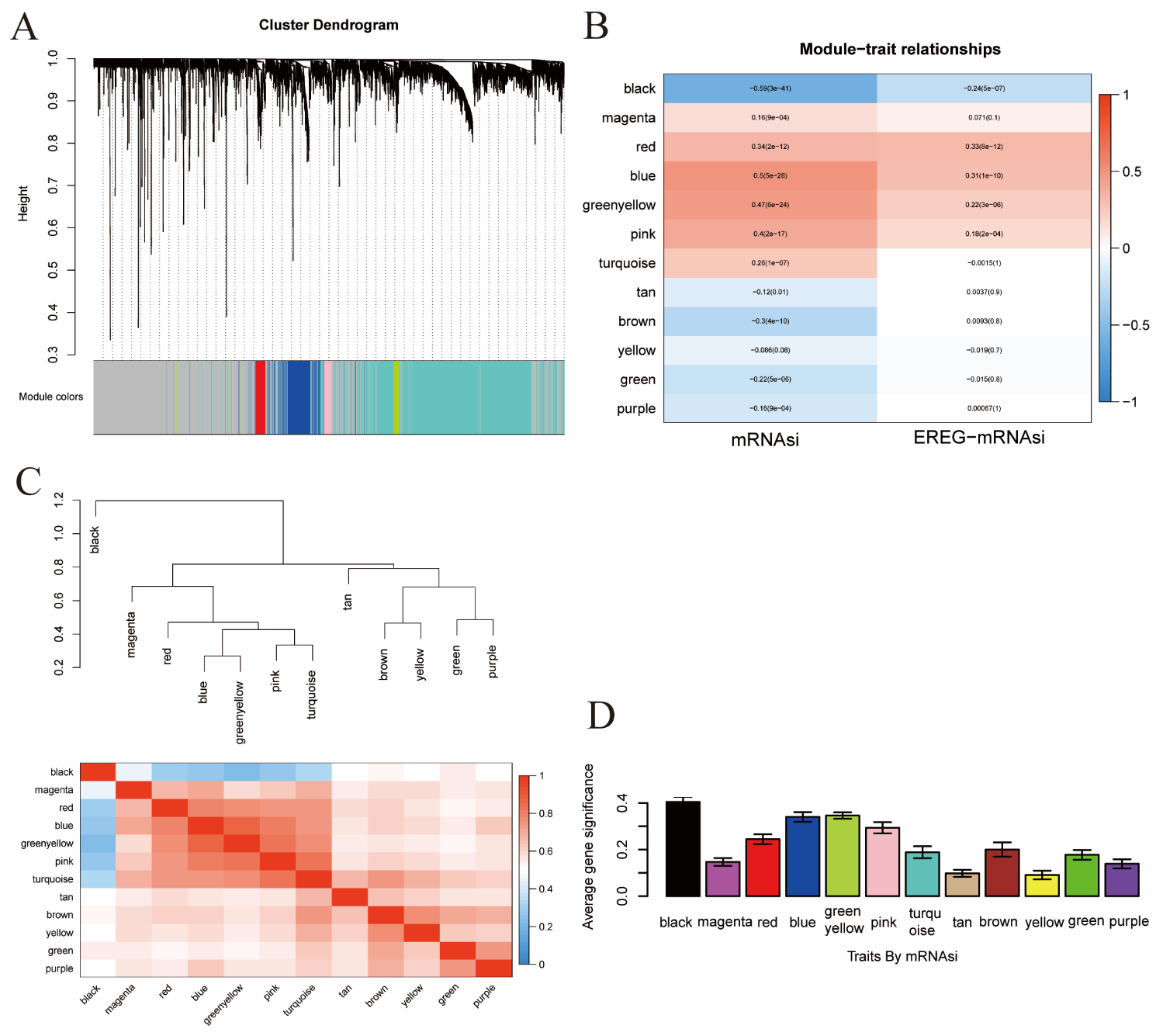


**Figure S1:** **Weighted gene co-expression network with DEGs between HCC and matched normal tissues.** **A** Identification of the co-expression modules in hepatocellular carcinoma. The branches of the cluster dendrogram correspond to the 12 different gene modules. Each piece of the leaves on the cluster dendrogram corresponds to a gene. **B** Correlation between the gene module and clinical traits including mRNAsi and EREG-mRNAsi. The correlation coefficient in each cell represented the correlation between the gene module and the clinical traits, which decreased in size from red to blue. The corresponding P-value is also annotated. **C** Correlation between modules and different traits. Absolute values of correlation coefficients between hepatocellular carcinoma-status and modules greater than 0.15 were considered as mRNAsi-related modules in HCC. **D** Average gene significance of twelve mRNAsi-related modules in HCC. The black, blue and green-yellow module were the most significant modules.


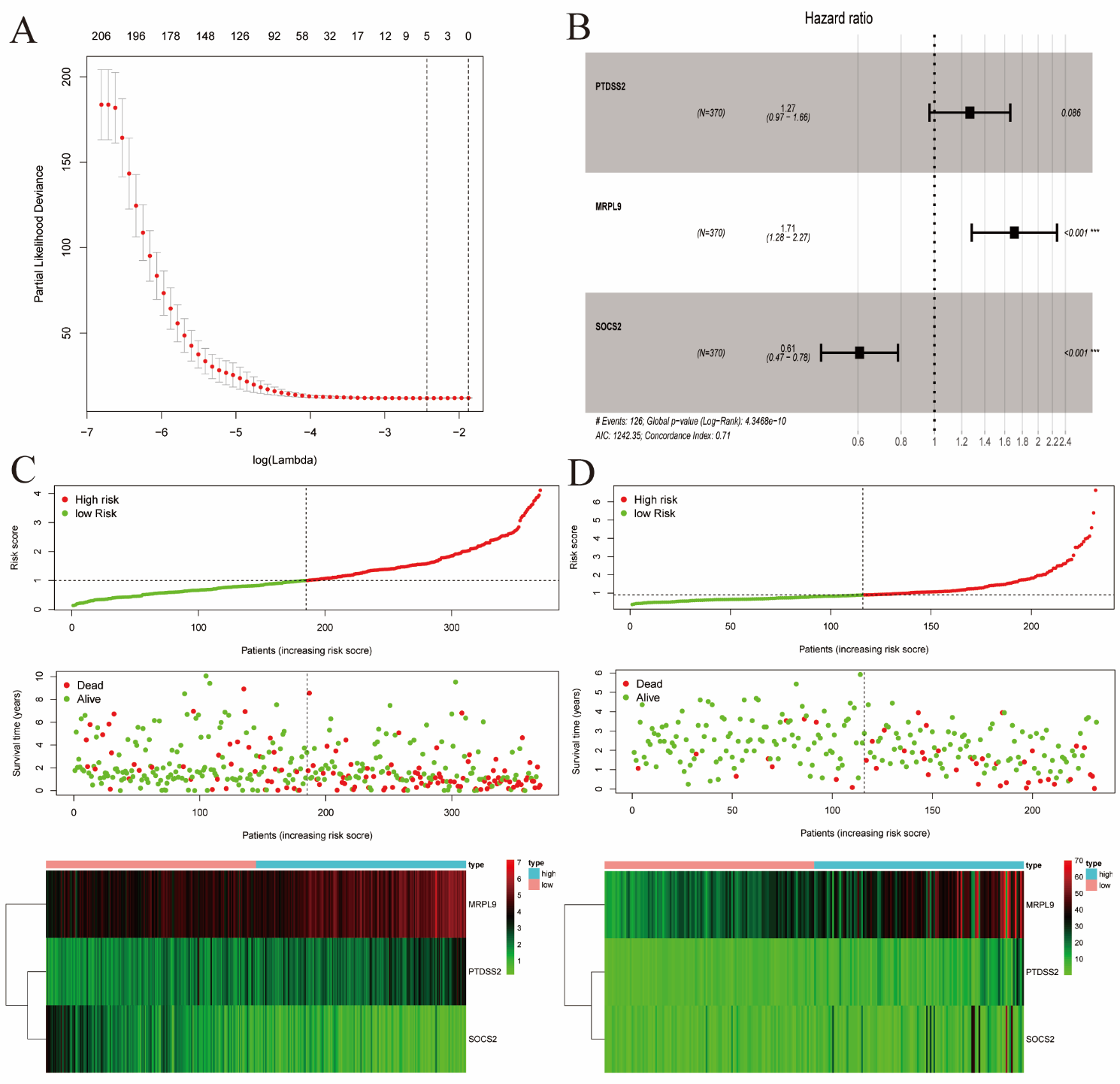


**Figure S2:** **The risk score based on the gene signature is a prognostic biomarker. A-B** The process of building the signature containing three gene correlated with overall survival in the training dataset. The hazard ratios (HR), 95% confidence intervals (CI) calculated by univariate Cox regression and the coefficients calculated by multivariate Cox regression using LASSO are shown. **C-D** Risk score distribution, survival overview and heatmap for patients in the TCGA (C) and ICGC (D) datasets assigned to high-and low-risk groups based on the risk score.


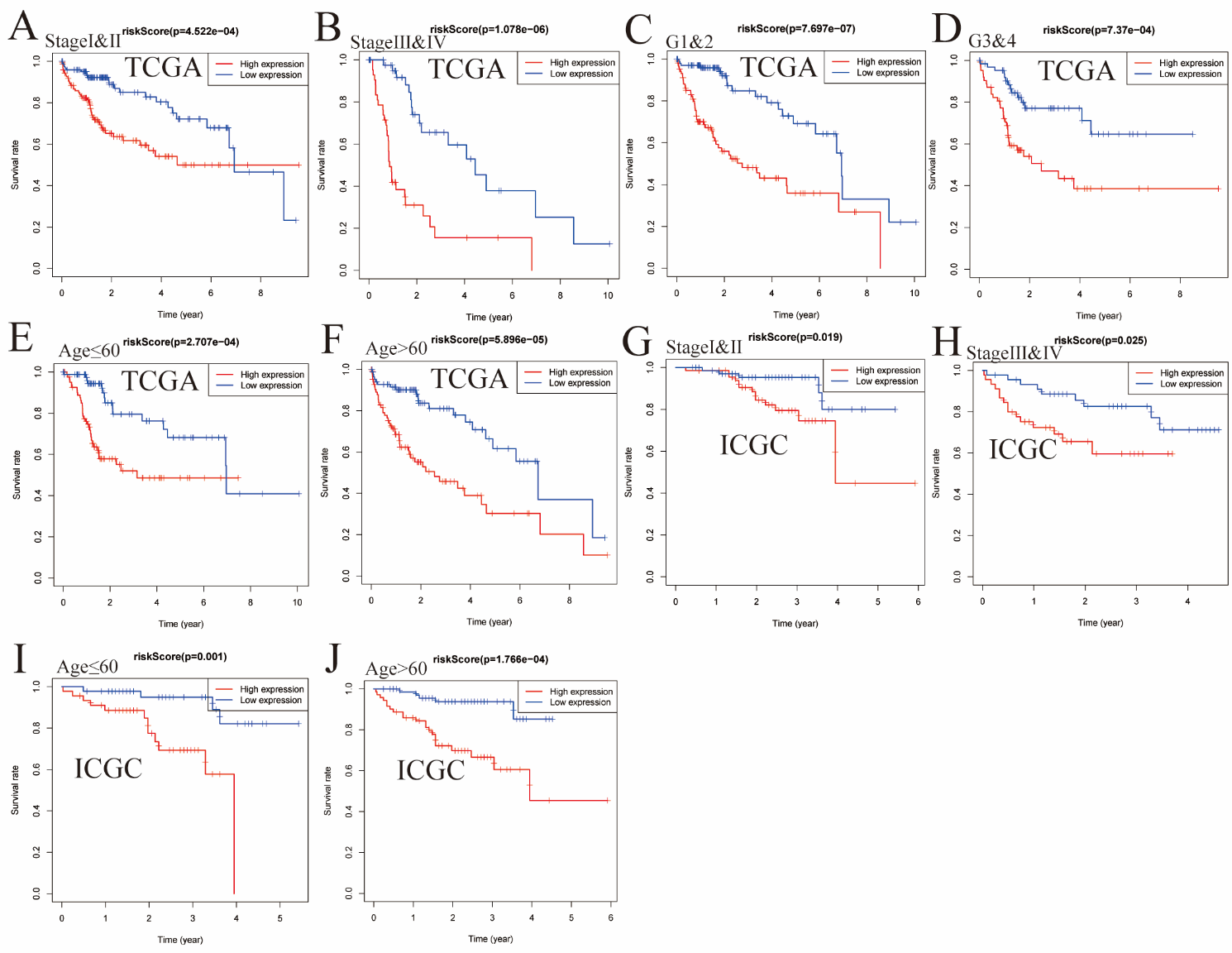


**Figure S3:** **Survival analysis of different subgroups divided by Clinicopathological factors.** The risk score based on the three-gene signature is a risk factor of overall survival in subgroups of stage I&Ⅱ (A), stage III&IV (B), grade l&2 (C), grade 3&4 (D), age <60 (E), age > 60 (F) in the TCGA and stage I&Ⅱ (G), stage III&IV (H), age <60 (I), age > 60 (J) in the ICGC.


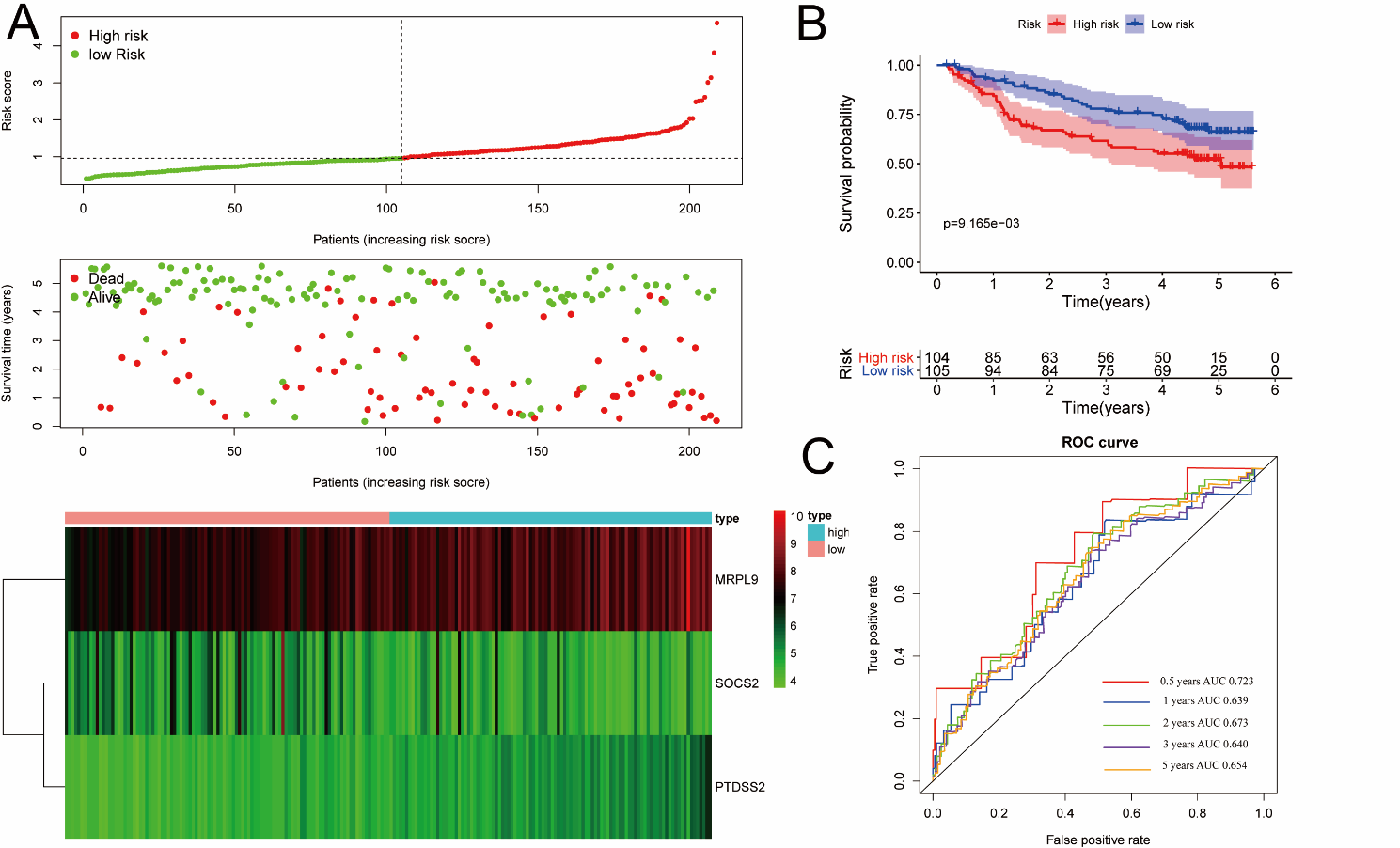


**Figure S4:** **An independent HCC cohort (****GSE14520) from the GEO database was used to verify the prognostic value of the risk score based on the three-gene signature. A** The risk score distribution, survival overview and heat map of patients assigned to high-risk and low-risk groups based on risk scores. **B** Kaplan-Meier curve of patients assigned to high- and low-risk groups according to risk scores. The prognosis of the high-risk group is poorer than that of the low-risk group. **C** The ROC curve shows the predictive efficiency of the risk score based on the gene signature on survival rate.


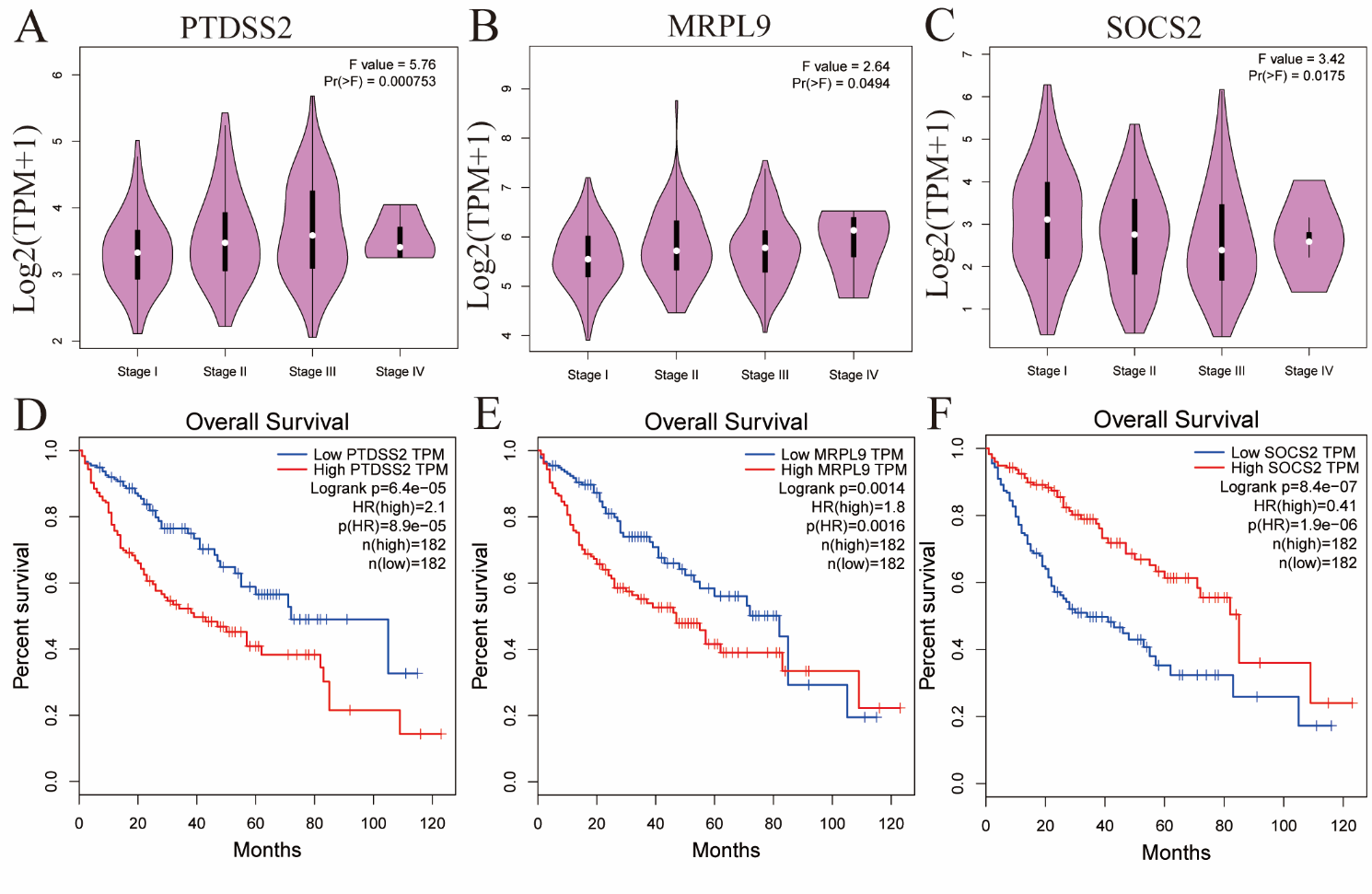


**Figure S5:** **Kaplan-Meier survival analysis with the mRNA expression of PTDSS2, MRPL9 and SOCS2. A-C** Comparison of three key genes expression among different tumor stages, respectively. **D-F** Overall survival was examined respect to gene expression of PTDSS2, MRPL9 and SOCS2, respectively. Survival analysis was performed in the TCGA HCC cohort. P-value was calculated using the log-rank test and is provided on the top right of each graph.


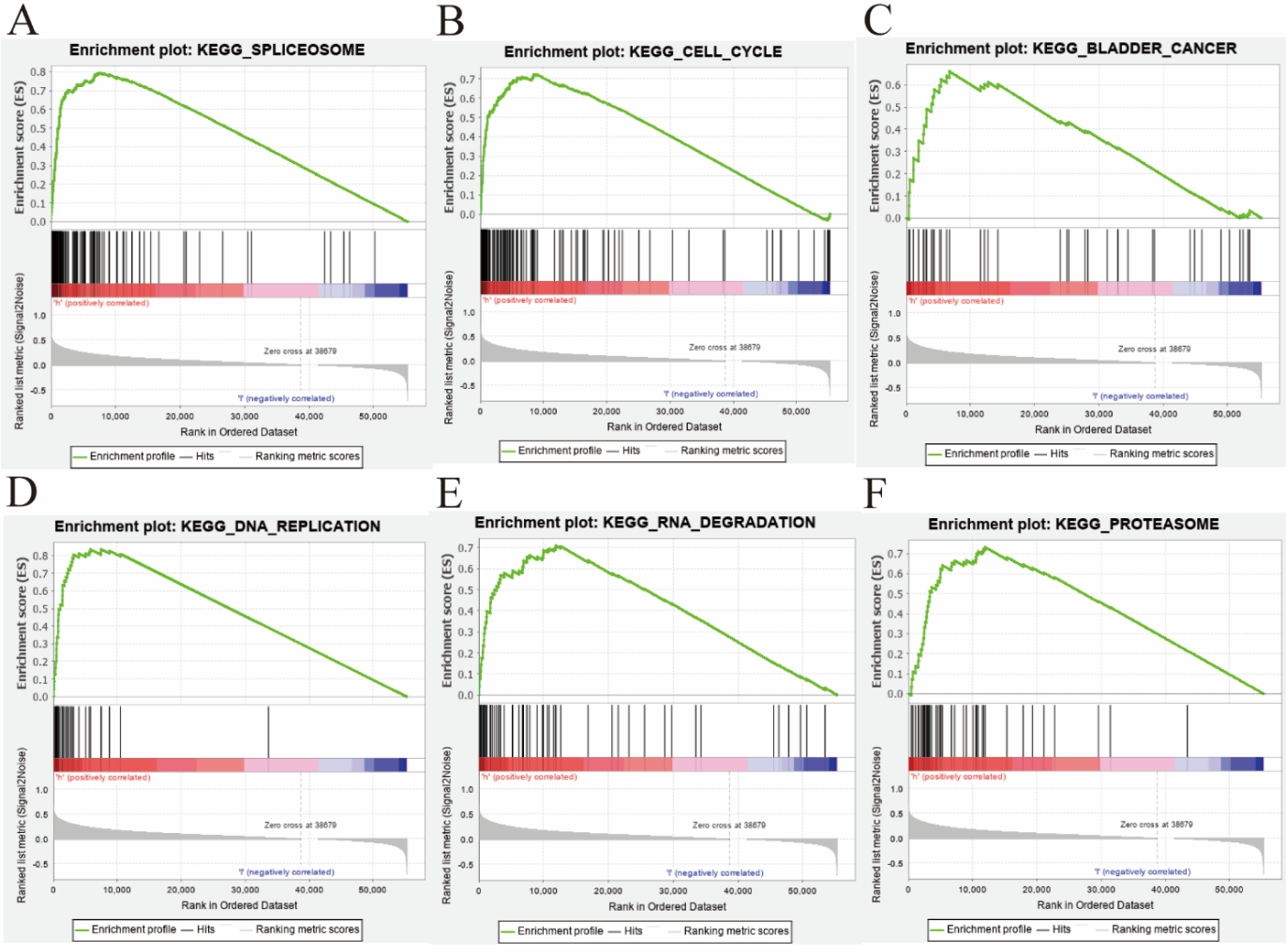


**Figure S6:** **Enriched pathways of up-regulated genes identified by GSEA in HCC.** NES, normalized enrichment score. Values are row-scaled to show relative expression. Blue and red are low and high levels, respectively.


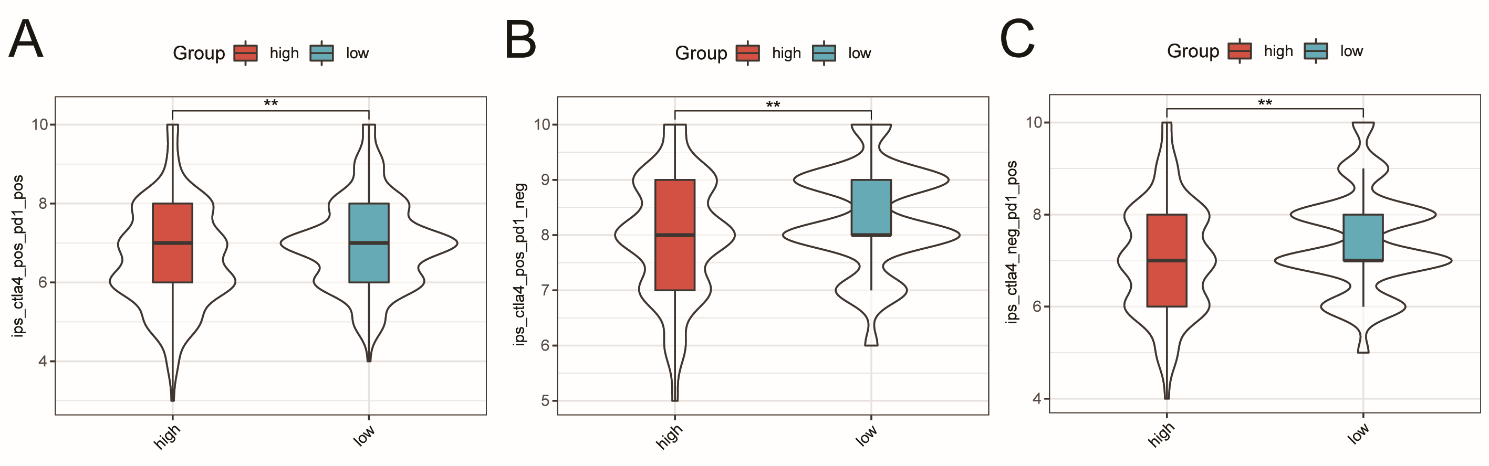


**Figure S7: The risk score predicts the benefit of immunotherapy.** The relative distribution of IPS between the high and low risk score groups in the TCGA cohort was compared, A: CTLA-4 (+) & PD-1 (+), B: CTLA-4 (+) & PD-1 (-), C: CTLA-4(-) & PD-1(+).

Supplementary Tables

**Table S1: Comparison of PTDSS2 expression values between HCC and matched adjacent non-tumor tissues in the GEO database**

| Dataset | P-value | Type | Nums | Mean | STD | IQR |
| --- | --- | --- | --- | --- | --- | --- |
| GSE22058 | 3.42E-08 | HCC | 100 | 9.132 | 0.5167 | 0.6774 |
|  |  | Adjacent | 97 | 8.79 | 0.2778 | 0.3748 |
| GSE25097 | 4.10E-12 | HCC | 268 | 1.098 | 0.5528 | 0.4637 |
|  |  | Adjacent | 243 | 0.8308 | 0.2452 | 0.221 |
| GSE36376 | 8.31E-55 | HCC | 240 | 7.51 | 0.5609 | 0.7222 |
|  |  | Adjacent | 193 | 6.734 | 0.2961 | 0.4191 |
| GSE14520 | 6.43E-11 | HCC | 225 | 4.806 | 0.4659 | 0.586 |
|  |  | Adjacent | 220 | 4.551 | 0.3226 | 0.416 |
| GSE63898 | 7.33E-14 | HCC | 228 | 5.374 | 0.5391 | 0.6926 |
|  |  | Adjacent | 168 | 5.058 | 0.2481 | 0.2999 |

**Table S2: Comparison of MRPL9 expression values between HCC and matched adjacent non-tumor tissues in the GEO database**

| Dataset | P-value | Type | Nums | Mean | STD | IQR |
| --- | --- | --- | --- | --- | --- | --- |
| GSE22058 | 3.63E-12 | HCC | 100 | 8.322 | 0.6632 | 0.8818 |
|  |  | Adjacent | 97 | 7.726 | 0.4349 | 0.4735 |
| GSE25097 | 2.52E-20 | HCC | 268 | 0.6263 | 0.3043 | 0.4005 |
|  |  | Adjacent | 243 | 0.4225 | 0.1479 | 0.179 |
| GSE36376 | 6.66E-50 | HCC | 240 | 7.284 | 0.5408 | 0.6938 |
|  |  | Adjacent | 193 | 6.59 | 0.265 | 0.378 |
| GSE14520 | 1.73E-76 | HCC | 225 | 6.517 | 0.6288 | 0.825 |
|  |  | Adjacent | 220 | 5.342 | 0.3972 | 0.4295 |
| GSE54236 | 1.09E-11 | HCC | 81 | 11.99 | 0.5202 | 0.595 |
|  |  | Adjacent | 80 | 11.47 | 0.3539 | 0.3633 |
| GSE64041 | 4.68E-11 | HCC | 60 | 9.303 | 0.4128 | 0.558 |
|  |  | Adjacent | 60 | 8.833 | 0.2722 | 0.3022 |
| GSE76427 | 2.04E-21 | HCC | 115 | 8.43 | 0.4167 | 0.52 |
|  |  | Adjacent | 52 | 7.742 | 0.325 | 0.4125 |

**Table S3: Comparison of SOCS2 expression values between HCC and matched adjacent non-tumor tissues in the GEO database**

| Dataset | P-value | Type | Nums | Mean | STD | IQR |
| --- | --- | --- | --- | --- | --- | --- |
| GSE22058 | 3.08E-34 | HCC | 100 | 9.021 | 1.16 | 1.546 |
|  |  | Adjacent | 97 | 11.15 | 0.7057 | 0.9459 |
| GSE25097 | 1.53E-70 | HCC | 268 | 1.5 | 1.236 | 1.489 |
|  |  | Adjacent | 243 | 5.096 | 2.219 | 3.214 |
| GSE36376 | 7.48E-49 | HCC | 240 | 7.694 | 0.7843 | 0.9062 |
|  |  | Adjacent | 193 | 9.134 | 0.9349 | 1.355 |
| GSE14520 | 6.54E-43 | HCC | 225 | 4.899 | 0.8121 | 0.949 |
|  |  | Adjacent | 220 | 6.383 | 1.162 | 1.742 |
| GSE10143 | 1.15E-10 | HCC | 80 | 13.64 | 0.7794 | 1.072 |
|  |  | Adjacent | 82 | 14.41 | 0.6302 | 0.954 |
| GSE46444 | 0.000363 | HCC | 88 | 6.732 | 0.592 | 0.7248 |
|  |  | Adjacent | 48 | 7.141 | 0.628 | 0.6624 |
| GSE63898 | 3.14E-62 | HCC | 228 | 6.206 | 1.233 | 1.697 |
|  |  | Adjacent | 168 | 8.552 | 1.073 | 1.394 |
| GSE64041 | 5.31E-06 | HCC | 60 | 7.479 | 0.5414 | 0.5897 |
|  |  | Adjacent | 60 | 7.943 | 0.522 | 0.4928 |
| GSE76427 | 7.03E-19 | HCC | 115 | 8.65 | 1.104 | 1.29 |
|  |  | Adjacent | 52 | 10.08 | 0.689 | 1.05 |

**Table S4: Enriched pathways of upregulated genes using GSEA in HCC**

| NAME | ES | NES | FDR q-val |
| --- | --- | --- | --- |
| Spliceosome | 0.794561 | 2.173327 | 0 |
| Cell_cycle | 0.722303 | 2.060145 | 0.002166 |
| Bladder_cancer | 0.660193 | 2.002992 | 0.004619 |
| DNA_replication | 0.836163 | 1.938743 | 0.007552 |
| RNA_degradation | 0.7094 | 2.05199 | 0.002265 |
| Proteasome | 0.7317 | 1.91865 | 0.008845 |

**Table S5:** **Potential therapeutic compounds from the CMap for the high-risk group,** **sorted by ascending P values**

| Rank | cMAP name | Mean | Enrichment | P | Percent non-null |
| --- | --- | --- | --- | --- | --- |
| 1 | Adiphenine | 0.758 | 0.965 | 0 | 100 |
| 2 | Trichostatin A | -0.433 | -0.285 | 0 | 75 |
| 3 | Tanespimycin | -0.422 | -0.306 | 0.00002 | 67 |
| 4 | Isoflupredone | 0.711 | 0.971 | 0.00004 | 100 |
| 5 | Prestwick-1103 | 0.675 | 0.905 | 0.00008 | 100 |
| 6 | Prestwick-692 | 0.602 | 0.871 | 0.00038 | 100 |
| 7 | Ly-294002 | -0.374 | -0.26 | 0.00038 | 63 |
| 8 | Monensin | 0.396 | 0.754 | 0.0005 | 66 |
| 9 | Merbromin | 0.602 | 0.806 | 0.00064 | 100 |
| 10 | 6-bromoindirubin-3'-oxime | 0.424 | 0.688 | 0.00066 | 71 |
| 11 | Biperiden | 0.567 | 0.801 | 0.0007 | 100 |
| 12 | Pyrvinium | -0.708 | -0.736 | 0.0007 | 100 |
| 13 | Sulfadimethoxine | 0.614 | 0.799 | 0.00078 | 100 |
| 14 | Diltiazem | -0.685 | -0.787 | 0.00084 | 100 |
| 15 | Nilutamide | 0.555 | 0.844 | 0.00088 | 100 |
